# Maternal and Neonatal Outcomes in Intrahepatic Cholestasis of Pregnancy

**DOI:** 10.3390/jcm12134407

**Published:** 2023-06-30

**Authors:** Roberta Granese, Gloria Calagna, Angela Alibrandi, Canio Martinelli, Paola Romeo, Roberto Filomia, Maria Immacolata Ferraro, Eleonora Piccione, Alfredo Ercoli, Carlo Saitta

**Affiliations:** 1 Department of Biomedical and Dental Sciences and Morphofunctional Imaging, University Hospital “G. Martino”, Via Consolare Valeria 1, Gazzi, 98100 Messina, Italy; 2 Obstetrics and Gynecology, “Villa Sofia Cervello” Hospital, University of Palermo, Via Trabucco 180, 90127 Palermo, Italy; 3 Department of Economics, Unit of Statistical and Mathematical Sciences, University of Messina, Via dei Verdi, 98166 Messina, Italy; 4 Department of Human Pathology in Adulthood and Childhood, University Hospital “G. Martino”, Via Consolare Valeria 1, Gazzi, 98100 Messina, Italy; 5 Department of Clinical and Experimental Medicine, University Hospital “G. Martino”, Via Consolare Valeria 1, Gazzi, 98100 Messina, Italy; 6 Family Counseling, ASP Messina, Via Catania 14, Sant’Agata di Militello, 98100 Messina, Italy; 7 Family Counseling, ASP Messina, Via Trento 8, Brolo, 98100 Messina, Italy

**Keywords:** intrahepatic cholestasis of pregnancy, liver disorder, serum bile acids

## Abstract

The aims of our study were to evaluate the maternal and fetal outcomes of intrahepatic cholestasis of pregnancy (ICP). In this observational, retrospective case–control study, we included all pregnant women who gave birth with a diagnosis of ICP between January 2010 and December 2020 at the Unit of Obstetrics and Gynecology, University Hospital of Messina. The data were compared with those from a control group of pregnant women who did not have ICP. One hundred twenty-nine and eighty-five patients were included, respectively, in the study and in the control group. There was a significant difference between the two groups in the incidence of hypothyroidism, thrombophilia, gestational diabetes, gestational hypertension, postpartum hemorrhage, and preterm delivery, which were more frequent in the ICP patients. No neonatal adverse events were recorded, although a significant difference in the meconium-stained amniotic fluid condition was noted. After a 24-month follow-up, 48/129 patients with ICP accepted to be reassessed by liver ultrasound, elastographic examination, and liver function blood tests. No patient showed signs of chronic liver disease. This study confirmed a higher probability of adverse short-term maternal outcomes in ICP pregnant patients, but a lower probability of adverse short-term fetal outcomes and the absence of a long-term maternal risk of chronic liver disease.

## 1. Introduction

Intrahepatic cholestasis of pregnancy (ICP) is the most common liver disease of pregnancy characterized by unexplained generalized pruritus associated with elevated serum bile acids and/or transaminases, presenting most commonly during the late second and third trimesters [1,2,3]. The literature on the topic reports an incidence of ICP ranging from 0.2% to 2% [4,5], with a wide variability based on ethnicity and geographic location and a higher frequency in South America and Northern Europe [5,6].

The pathogenesis of ICP is manifold and still has some unclear aspects: genetic, hormonal, and exogenous factors seem to be involved [7]. A genetic predisposition interacting with the metabolites of reproductive hormones may interfere with bile secretory mechanisms [2]. Mutations of the hepatobiliary transport protein-multidrug resistance protein 3 (MDR3) involved in the biliary secretion of phospholipids have an important role [8]. MDR3 mutations occur in approximately 16% of all ICP cases, and the type of mutation is correlated with the severity of the disease [9]. Another transport protein contributing to the development of ICP is the multidrug resistance-related protein 2 (MRP2). However, this mutation was highlighted only in a population of South American women, never among Caucasians [10]. In recent studies, an interesting relationship was found between ICP and a mutation in the BSEP gene [11]. In Caucasian patients affected by ICP, rare mutations in the FIC1 gene (ATP8B1), expressed in the bile duct membrane, and in the FXR gene (NR1H4) were also detected [12,13]. Those mutations may be due to steroid hormones or their metabolites. These data have been confirmed by the more frequent incidence of ICP in multiple gestations or in patients treated with oral contraception. Moreover, the more frequent diagnosis of ICP in the third trimester of pregnancy, when the hormones reach the highest peak, and the resolution of the disease after birth, when the hormones decrease, also provide a strong confirmation of the correlation considered [14]. However, how sex hormones contribute to the development of ICP has not been clearly understood. Moreover, other risk factors have been identified, such as in vitro fertilization, advanced maternal age, and hepatitis C infection [4,15].

As already reported, the disease resolves spontaneously after delivery [16], and the likelihood of recurrence during a subsequent pregnancy is about 60% [17].

The diagnosis is based on the presence of specific signs and symptoms during pregnancy: elevated serum bile acids (in the absence of other hepatobiliary disease) and itching without skin rash, usually involving the palms and the soles of the feet. Based on literature data, a bile acid concentration of 10 μmol/L or greater indicates ICP in pregnancy. Other possible manifestations of ICP include mild jaundice, steatorrhea, and hypocholic stools. 

The relevance of this pathology is mainly due to possible associated obstetrical and fetal complications, such as preterm labur, fetal asphyxia, meconium-stained amniotic fluid, and even stillbirth [3,18,19,20]. Most studies agree to consider 10μmol/L as a diagnostic cut-off for total bile acids; a recent classification identified three forms of ICP gravity: mild (10–39.9 μmol/L), moderate (40–99.9 μmol/L), and severe (≥100 μmol/L) [8]. It was documented that an increased bile acid peak concentration (in particular ≥40 μmol/L) is associated with higher rates of adverse perinatal outcomes [21,22,23,24]. Thus, in the last few years, many studies focused on the significance of the association between elevated bile acid concentrations and pregnancy complications, in order to establish better management strategies [25,26]. Recent data demonstrated that the risk of adverse neonatal events is greater as the value of bile acid increases, highlighting a positive linear correlation between the bile acid level and poor pregnancy outcomes [9,20,24,27]. However, no method of fetal monitoring has been shown to reduce the risk of stillbirth [21,24,28]. 

In the absence of dedicated guidelines, the principal recommendations in the case of ICP are careful surveillance and evaluation of the timing of delivery, with the possibility of early iatrogenic delivery, according to the serum bile acids concentration, in order to minimize adverse fetal and neonatal outcomes [24]. Moreover, ursodeoxycholic acid is commonly used for the treatment of ICP, as it has an anti-inflammatory action and can reduce the elevation of the serum bile acid concentration in the fetus, probably by upregulating the placental bile acid export [29,30,31]. However, there is no clear literature evidence on its benefits with respect to the reduction of adverse perinatal outcomes in women with ICP [24], and larger-size samples are required to achieve reliable data [24,32,33,34].

The aims of this study were to evaluate the maternal and fetal outcomes of the ICP cases managed in our Hospital Unit, in comparison to control pregnancies.

## 2. Materials and Methods

This population-based study was conducted between January 2010 and December 2020 at the Unit of Obstetrics and Gynecology of the A.O.U. “G. Martino” of Messina in collaboration with the Unit of Clinical and Biomolecular Hepatology. This was an observational, retrospective, case–control study. The study was approved by the Local Ethics Committee and was written following the guidelines of observational studies (STROBE). We included all pregnant women managed at our Unit of Obstetrics and Gynecology who gave birth with a diagnosis of ICP and who signed an informed consent for data collection for research purposes at admission. The data were compared with those from a control group of pregnant women, who had given birth in the same study period and signed an informed consent. For this purpose, we selected patients without differences for parity, previous caesarean deliveries, familiarity with cholestasis, and previous ICP, compared to the study group. We excluded pregnant women who presented symptoms of cholestasis and normal laboratory tests, pregnancies interrupted before the 24th week, and women who did not sign the informed consent for study data collection. For each patient, we collected personal data, medical family history, past medical history, obstetric anamnestic data, neonatal data, laboratory tests data, also including those from liver function tests and markers for human immunodeficiency virus (HIV), data regarding venereal disease research laboratory test (VDRL), hepatitis B and C (HBV and HCV) viruses, TSH values, and data on congenital mutations of factors II and V Leiden, prot C, prot S, and ATIII, reported in the medical records of all the women involved in the research.

The patients included in the study were contacted again 24 months after delivery to carry out a follow-up visit (ultrasound (US) examination of the liver, hepatic elastography, and a dedicated evaluation of liver function tests, in particular, of full blood count, transaminase, gamma-GT, alkaline phosphatase, and bilirubin values). The patients with ICP were all treated with ursodesoxycholic acid (UDCA) at a daily dosage of 900 mg. 

### Statistical Analysis

The numeric variables are described as mean ± standard deviations (SD); the categorical variables are described as absolute and percentage frequency. Since most analyzed variables were not normally distributed, as verified through the Kolmogorov–Smirnov test, the non-parametric approach was used for statistical analysis. In order to compare patients with and without ICP, the Chi-square test, the likelihood ratio test, or the Fisher’s exact test (when appropriate) for categorical variables and the Mann–Whitney test for numerical parameters, were applied. The Wilcoxon test was used to compare two time points (pre-therapy vs. post-therapy) for bile acids, bilirubin, GGT, GOT, GPT, alkaline phosphatase, platelets, and PT parameters. The McNemar test was applied to compare two time points (pre-therapy vs. post-therapy) for itching, which was a dichotomous variable (yes or no). The odds ratio (with a 95% confidence interval and statistical significance) was calculated to quantify the risk in pregnant women suffering from cholestasis of developing obstetric pathologies such as hypertension, diabetes, meconium-stained amniotic fluid, threatened preterm labor, hypothyroidism, and newborn complications (including the necessity of respiratory assistance). A *p*-value < 0.05 was considered statistically significant. SPSS software for Windows (version 22.0) was used for statistical processing. 

## 3. Results

**Figure 1:**
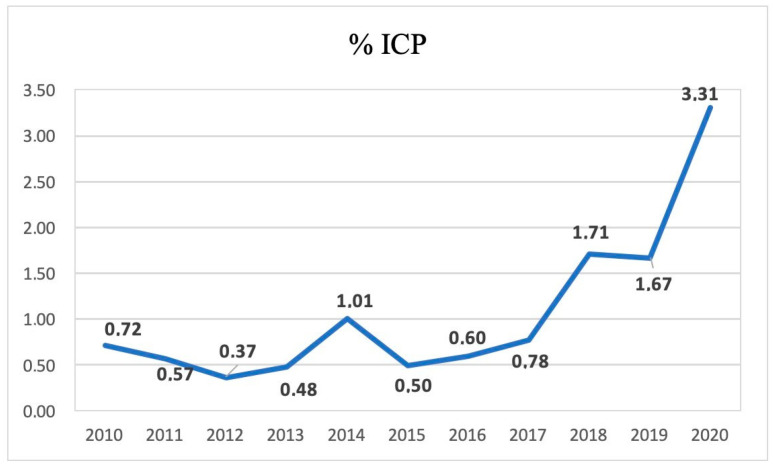
Trend of the 10-year ICP incidence.

Between January 2010 and December 2020, a total of 13,641 women gave birth at the Unit of Obstetrics and Gynecology, University Hospital of Messina; among these, ICP was diagnosed in 150 women, with an overall incidence of 1.1% and an increasing trend over the years (Figure 1). 

Among the 150 ICP patients, 129 accepted to participate in the study, signing the informed consent. As requested in the design of the study, 85 pregnant women who did not have ICP were considered as a control group. Ninety-five percent of the women studied in both groups were Italian. The median gestational age at delivery was 38 + 2 days. All subjects with ICP presented with a mild or moderate increase of bile acid levels, except for one woman with severe ICP (bile acids > 100 μmol/L). A comparison between the data of the study and the control groups is reported in Table 1.

For patients affected by ICP, the values of GOT, GGT, GPT, and alkaline phosphatase were significantly higher compared to those for patients of the control group (Table 1). There was a statistically significant difference between the two groups in the incidence of some pathologies. i.e., hypothyroidism (*p* < 0.001), thrombophilia (for congenital mutations of factors II, V Leiden in homozygous or heterozygous state associated with low levels of prot S, prot C, or AT III) (*p* = 0.011), gestational diabetes (*p* < 0.001), gestational hypertension (*p* < 0.001), threatened preterm labor (*p* = 0.001), postpartum hemorrhage (*p* = 0.017), and spontaneous preterm delivery (*p* = 0.001), which were more frequent in ICP patients (Table 2). 

Moreover, a significant difference in the timing of delivery was highlighted: women affected by ICP delivered most frequently between 32 and 37 weeks (16.3% versus 9.5%) of gestational age, in contrast to women of the control group, who delivered mostly between 38 and 40 weeks of pregnancy (*p* = 0.001).

Referring to the ICP group, all patients had a physiological course of puerperium. No positive markers for hepatitis, syphilis, and HIV were found. A chromosomal abnormality screening was negative in all patients. US evaluations during the trimesters of pregnancy did not show any pathology, and all the pregnancies were correctly dated; however, a statistically significant difference in fetal weight estimation in the third trimester scan was noted, reporting a lower weight in patients with ICP (*p* = 0.002). This finding was confirmed at birth, as the children born to the study group patients had a significantly lower weight than the children born to mothers without ICP (*p* = 0.007) (Table 1). 

No neonatal adverse events were recorded (such as neonatal intensive care, peri-partum asphyxia, or long-term care), although a significant difference in the meconium-stained amniotic fluid condition was noted (*p* < 0.001). There was also a difference in the modality of delivery: the study group patients had a tendency of a higher percentage of caesarean sections (Table 2), even if not statistically significant; however, the percentage of caesarean sections during labor due to alterations of cardiotocography was statistically significant (*p* = 0.005).

To quantify the risk for women suffering from ICP of developing some pathological conditions, the odds ratio was calculated for gestational diabetes, gestational hypertension, preterm delivery, hypothyroidism, thrombophilia, neonatal complication, and meconium-stained amniotic fluid, which was statistically significant in all cases except for neonatal complications (Table 3). 

To assess the effects of the treatment with UDCA in patients with ICP, the Wilcoxon test was used, and this test showed significant reductions for bile acids, GOT, GPT, and GGT, besides a clear decrease in itching (98.4% of cases; *p* = 0.001) (Table 4). 

After a 24-month follow-up, 48/129 patients with ICP accepted to be reassessed at the Unit of Clinical and Biomolecular Hepatology. All women underwent liver US, elastographic examination, and liver function blood tests. At US examination, 12 (25%) patients had mild liver steatosis, 2 (4.2%) had moderate steatosis, 1 (2.1%) had a choledochus stenosis and mild dilation of the intrahepatic biliary tract. No patient had alterations of the hepatic parenchyma and/or of the portal vascularization indicative of chronic liver disease. Gallbladder stones, a condition that could be associated with cholestasis in pregnancy, was not detected in any patient. The liver elastographic examination showed mean values of hepatic stiffness of 4.63 Kpa (S.D. 1.4 Kpa); in only seven patients were the liver stiffness values > 6 Kpa (range 6–7.5). The liver function blood tests (GOT, GPT, GGT, ALP, bilirubin, and PLT count) were within the normal range in all cases.

## 4. Discussion

In recent years, several studies have been carried out on ICP, indicating the greater attention being paid to this pathology. Researchers have focused on two principal aspects: 1. the role of increased serum total bile acids as a diagnostic criterion and as a predictive factor of maternal and fetal complications; 2. the importance of ursodeoxycholic acid treatment in the management of the pathology. 

Numerous data are available in the literature on the association of maternal comorbidities (such as pre-eclampsia, gestational diabetes, etc.) with adverse perinatal outcomes in women with increased serum bile acid concentrations > 40 μmol/L [23,24,27,35,36,37]. A recent meta-analysis and the RCOG Green-top guidelines showed a significantly increased risk of stillbirth only for women with serum total bile acids of 100 μmol/L or more [21,24]. In particular, an increased risk ranging from 0.13% in women affected by mild ICP to 3.44% in women affected by severe ICP was found [21].

The mechanism by which bile acids act seems to be related to an action on several levels. After crossing the placenta, they may influence fetal cardiomyocytes, inducing arrhythmias, cause distress symptoms at the respiratory level, and interfere in the chorionic vessels and/or in the myometrium, inducing preterm birth or intrauterine death [38,39,40]. 

Our study’s results align with literature data that highlight a correlation between ICP and several adverse maternal and fetal outcomes, such as an increase in preterm deliveries (a 15-time higher risk compared to patients without ICP), post-partum hemorrhage, and emergency caesarean section risk. 

In particular, we found a higher incidence of caesarean section for changes in the cardiotocographic tracing and also an increased risk of meconium-tinged amniotic fluid (a 35-time higher risk compared to patients without ICP). However, at birth, a physiological Apgar score was always recorded in the ICP study group; this fact could also be explained by the fact that in our sample almost all cases were of the mild or moderate form of ICP. 

Based on these data, the management of the time of delivery takes on particular importance. Although some studies identified 36 weeks as the optimal delivery point to prevent stillbirth or neonatal death for singleton pregnancies, a stratification of the patients according to disease severity was not considered, as neither was the additional neonatal morbidity secondary to prematurity [17,41]. Successive experiences and systematic reviews pointed out that the evidence was insufficient to support the practice of active management, mainly preterm, for all ICP patients [28]. The Society for Maternal–Fetal Medicine suggested a different management based on whether the peak bile acid was >100 μmol/L or <100 μmol/L, recommending offering the possibility of delivering at 36 0/7 weeks of gestation in the first case and between 36 0/7 and 39 0/7 weeks of gestation in the second case [31]. Finally, the 2022 RCOG guidelines recommend planning birth according to the bile acid values. To women with peak bile acids between 19 and 39 μmol/L (mild ICP) and no other risk factors, it is suggested to plan birth by 40 weeks of gestation; to women with peak bile acids between 40 and 99 μmol/L (moderate ICP) and no other risk factors, it is suggested to plan birth at 38–39 weeks of gestation; and to women with peak bile acids equal to or higher than 100 μmol/L (severe ICP), it is suggested to plan birth at 35–36 weeks of gestation [24]. 

In our hospital, the management of patients with ICP consists in hospitalization if the therapy with ursodeoxycholic acid at home does not lower the value of the bile acids or if the itching becomes worse. This way, patients can be better monitored prior to establishing the most suitable time for childbirth on the basis of laboratory tests. Induction of labor is decided according to the bile acid values. If a pregnant woman is affected by mild and moderate ICP (as the women included in our study, except one), we do not induce the delivery before 37 weeks. Different considerations must be made for severe ICP cases (bile acids =/>100 μmol/L), generally induced between 36 0/7 and 36 7/7 weeks, mainly considering that the risk of stillbirth seems to be increased [21]. The presence of risk factors or co-morbidities (i.e., gestational diabetes and/or pre-eclampsia and/or multifetal pregnancy) further increases the risk of fatal outcomes; hence, labor could be induced even before 36 0/7 weeks of gestation, after pulmonary maturity induction [24].

Most patients of our study sample gave birth between the 32nd and the 37th week of gestation due to cardiotocographic changes that led to an emergency caesarean section or to the spontaneous onset of labor. In cases in which pregnancy went beyond the 37th week, no significant adverse fetal outcomes were recorded. 

The data of our study sample support a conservative behavior for mild and moderate ICP, as the incidence of severe maternal and neonatal complications is demonstrated to be low, and, hence, the induction of labor before the 37th week is not justified. 

The choice of the type of birth (vaginal vs. cesarean section delivery) should depend on obstetric indications [16].

Another emerging piece of data of our research, also confirmed by other authors [22], is the enhanced risk of developing gestational diabetes, gestational hypertension, hypothyroidism, and thrombophilia for ICP cases compared to control pregnancies, and this difference was statistically significant. In particular, patients with gestational hypertension showed a 10-time higher risk of developing ICP compared to non-hypertensive patients; in women affected by hypothyroidism, the risk was 11 times higher; thrombophilic patients and diabetic patients had, respectively, a 9- and 5-time greater risk of developing ICP. An explanation of this increased incidence could be found in the action of the bile acids at the “placental level”. Moreover, the study group of Martineau et al. showed that the bile acids play a role also in glucose homeostasis through the activation of specific nuclear receptors [42].

It is important to underline that the overall incidence of ICP in our study sample was 1.09%, with an increasing trend over the years. This datum seems to underestimate the extent of the phenomenon but, in our opinion, could be the result of an initial lack of knowledge of the problem and, therefore, of less attention to its management. This could be a bias in the report of the incidence of ICP over the years. In particular, at the beginning of our data collection, the incidence of ICP was 0.72, reaching, at the end, a value of 3.31 (Figure 1). 

Focusing on the therapeutic approach, our study group was treated with oral ursodeoxycholic acid, at a dosage of 300 mg 2–3 times per day (or 10–16 mg/kg/day), as suggested by literature data [16]. 

This drug is the most used treatment for intrahepatic cholestasis of pregnancy, and recent data concluded that its use is safe and effective [16,31,43]. However, there is no consensus on this therapy, as its benefits and mechanism of action are not completely understood [24,38]. In a recent meta-analysis, two important observations on this topic were made: ursodeoxycholic acid treatment, in women with ICP, reduced the risk of preterm birth and, when considering only randomized controlled trials, ursodeoxycholic acid was associated with a reduction in stillbirth in combination with preterm birth, providing evidence for the clinical benefits of an antenatal ursodeoxycholic acid treatment in these patients [44]. For this reason, ursodeoxycholic acid could be suggested for all women with onset of ICP before 37 weeks of gestation to reduce these risks [21].

ICP may also reduce the absorption of vitamin K, leading to an increase in prothrombin time, which may result in postpartum bleeding; therefore, some authors suggested the administration of vitamin K at the dose of 10 mg [16].

Finally, in our study sample, after a 24-month follow-up, no changes in blood chemistry were reported, confirming the resolution of the maternal disease after childbirth. In addition, at US examination and hepatic elastography, no changes were highlighted, with the exception of a few cases of mild–moderate steatosis, thus confirming a favorable long-term maternal outcome. Although this is a reassuring observation, a study bias must be considered: our sample only consisted of patients diagnosed with mild and moderate cholestasis (bile acids < 100 μmol/L). 

The data present in the literature suggest discussing women care with an hepatologist in all cases in which women develop ICP in the first trimester in order to consider further investigation and treatment options. This is recommended as, in these cases, it is more frequent to have an association with a genetic predisposition or an alternative or additional diagnosis. Moreover, a strong monitoring in the post-natal period has to be considered for all women who do not show a resolution of itching and present abnormal laboratory values after giving birth [24]. 

Instead, no alarm should be raised for patients affected by cholestasis of mild and medium entity developed in the third trimester of pregnancy, if an early diagnosis and all the appropriate therapeutic measures are undertaken. 

## 5. Conclusions

This study revealed a trend of a higher incidence of ICP in the last ten years in our hospital in Southern Italy. It also confirmed a higher probability of adverse short-term maternal outcomes but a lower probability of adverse short-term fetal outcomes. Moreover, the absence of a long-term maternal risk of chronic liver disease was highlighted. 

## Figures and Tables

**Table 1:** Comparison between patients with and without ICP; numeric variables.

	Pts with ICP (n. 129) Mean ± SD	Pts without ICP (n. 84) Mean ± SD	*p*
Age (years)	34.08 ± 6.11	32.97 ± 6.92	0.220
Pre-pregnancy weight (kg)	62.53 ± 14.10	60.12 ± 8.57	0.721
Post-pregnancy weight (kg)	75.35 ± 15.92	72.80 ± 8.37	0.578
Height (cm)	161.05 ± 14.31	162.61 ± 5.94	0.434
Direct bilirubin (mg/dL)	0.90 ± 0.72	0.67 ± 0.17	0.149
Indirect bilirubin (mg/dL)	0.47 ± 0.21	0.33 ± 0.01	0.295
PLT (mmc)	227.73 ± 67.92	208.11 ± 52.60	0.199
GOT (U/L)	134.56 ± 47.81	17.56 ± 5.53	**<0.001**
GGT(U/L)	67.11 ± 34.14	32.10 ± 11.23	**<0.001**
GPT (U/L)	78.72 ± 21.17	17.32 ± 12.54	**<0.001**
Alkaline phosphatase (U/L)	177.69 ± 75.90	89.12 ± 13.93	**<0.001**
PT (%)	83.84 ± 46.4	91.23 ± 42.21	0.183
Neonatal weight (kg)	2961.88 ± 525.13	3156.31 ± 508.19	**0.007**
Apgar 1′	9.1 ± 1.21	8.62 ± 1.82	0.057
Apgar 5′	9.78 ± 0.53	9.55 ± 1.6	0.971

**Table 2:** Comparison between patients with and without ICP; categorical variables.

	Pts with ICP (n. 129) N (%)	Pts without ICP (n. 84) N (%)	*p*
Hypothyroidism	25 (19.4)	1 (1.2)	**<0.001**
Thrombophilia	13 (10.1)	1 (1.2)	**0.011**
Gestational Diabetes	27 (20.9)	4 (4.8)	**<0.001**
Gestational Hypertension	27 (20.9)	2 (2.4)	**<0.001**
Threatened Preterm Labor	20 (15.5)	1 (1.2)	**0.001**
Post-Partum Hemorrhage	8 (6.5)	0 (0.0)	**0.017**
Caesarean section	65 (53.3)	34 (40.5)	0.071
Spontaneous pre-term delivery	21 (16.3)	8 (9.5)	**0.001**
Meconium-stained amniotic fluid	60 (46.5)	2 (2.4)	**0.001**

**Table 3:** Association between Intrahepatic Cholestasis of Pregnancy and obstetric and neonatal outcomes.

	OR	95% CI	*p*
Preterm delivery	15.229	2.003–115.791	**0.009**
Gestational Hypothyroidism	11.700	3.482–39.317	**<0.001**
Gestational Hypertension	10.853	2.507–46.987	**0.001**
Thrombophilia	9.302	1.193–72.499	**0.033**
Gestational Diabetes	5.294	1.780–15.748	**0.005**
Neonatal Complications	2.677	0.294–24.379	0.382
Meconium-stained amniotic fluid	35.652	8.407–151.192	**<0.001**

**Table 4:** Comparison between pre- and post-treatment mean values in patients with intrahepatic cholestasis of pregnancy.

	Pre-Treatment (Mean ± SD)	Post-Treatment (Mean ± SD)	*p*
Bile acid (μmol/L)	44.37 ± 19.80	20.11 ± 13.72	**0.005**
Direct bilirubin (mg/dL)	0.90 ± 0.72	0.82 ± 0.68	0.359
Indirect bilirubin (mg/dL)	0.47 ± 0.21	0.54 ± 0.40	0.125
Alkaline phosphatase (U/L)	177.69 ± 75.90	211.08 ± 111.92	0.655
PLT (mmc)	227.73 ± 67.92	311.02 ± 41.43	0.113
GOT(U/L)	134.56 ± 47.81	52.51 ± 36.10	**<0.001**
GGT(U/L)	67.11 ± 34.14	21.44 ± 14.60	**0.004**
GPT(U/L)	78.72 ± 21.17	26.91 ± 11.77	**<0.001**
PT (%)	83.84 ± 46.45	86.20 ± 44.87	0.267

## Data Availability

The data that support the findings of this study are available from the corresponding author upon reasonable request.
